# Human responses to social-ecological traps

**DOI:** 10.1007/s11625-016-0397-x

**Published:** 2016-09-29

**Authors:** Wiebren Johannes Boonstra, Emma Björkvik, L. Jamila Haider, Vanessa Masterson

**Affiliations:** Stockholms Universitet, Stockholm, Sweden

**Keywords:** Social-ecological traps, Sociology, Responses, Typology, Primary production, Rural development

## Abstract

Social-ecological (SE) traps refer to persistent mismatches between the responses of people, or organisms, and their social and ecological conditions that are undesirable from a sustainability perspective. Until now, the occurrence of SE traps is primarily explained from a lack of adaptive capacity; not much attention is paid to other causal factors. In our article, we address this concern by theorizing the variety of human responses to SE traps and the effect of these responses on trap dynamics. Besides (adaptive) capacities, we theorize desires, abilities and opportunities as important additional drivers to explain the diversity of human responses to traps. Using these theoretical concepts, we construct a typology of human responses to SE traps, and illustrate its empirical relevance with three cases of SE traps: Swedish Baltic Sea fishery; amaXhosa rural livelihoods; and Pamir smallholder farming. We conclude with a discussion of how attention to the diversity in human response to SE traps may inform future academic research and planned interventions to prevent or dissolve SE traps.

## Introduction

The concept of ‘traps’ refers to situations of mismatch between the responses of people, or organisms and the social and ecological conditions that trigger these responses (Platt 1973; Costanza 1987). Essential for a trap situation is that the mismatch is persistent and self-reinforcing (Boonstra and de Boer 2014). A trap, in other words, refers to an adverse situation which is persistent, because the behavioural responses it triggers contribute to the reproduction of the adversity.

The trap concept is used across the sciences (Boonstra and Hanh 2015; Haider 2015). In biology, it refers to the *“*maladaptive behaviors*”* of species to rapidly changing environmental conditions (Schlaepfer et al. 2002: 474). In the social sciences, traps refer to situations in which people fail to avoid outcomes that are psychologically *“*unpleasant or lethal*”* (Platt 1973: 641), situations in which people cannot realize cooperation (Rothstein 2005), or when individuals or groups of people suffer from chronic poverty (Azariadis 2005). Social-ecological (SE) traps refer to rigid and inert behavioural responses that reinforce unsustainable outcomes (Cinner 2011; Enfors 2013; and Steneck et al. 2011).

In the sustainability literature, the occurrence of SE traps has so far primarily been explained by lack of ‘adaptive capacity’ (Carpenter and Brock 2008; Scheffer and Westley 2007), or the synonymous term ‘adaptability’. The terms refer to the capacity to learn and use knowledge to adjust behaviour according to changes in social-ecological conditions (Folke et al.  2010). These explanations typically provide no other qualification than between adaptation and maladaptation, and do not consider other additional causes besides adaptability that trigger human response. From a social scientific point of view, it is possible and necessary to provide more nuanced distinctions between the types of human response, which, in turn, can also help to identify more causes of SE traps and offer a wider spectrum of intervention strategies.

The objective of this article is to address the above concerns and to make the concept of SE traps more useful for interdisciplinary science. To this purpose, we introduce a simple theoretical model that engages three interrelated concepts: desires, abilities, and opportunities, to help theorize the variety of human responses to trap situations. Based on this theoretical framework, we construct a typology of human responses to SE traps. The empirical relevance of both the framework and the typology is then illustrated with three cases of SE traps: Swedish Baltic Sea fishery; amaXhosa rural livelihoods; and Pamir smallholder farming. We conclude with a discussion of how attention to the diversity in human response to SE traps may inform future academic research and planned interventions to prevent or dissolve SE traps.

## A simple (but not too simple) model of human response

The idea of mismatch between behavioural responses and social or ecological conditions belongs to a long line of thinking in the social sciences. The early sociologists, such as Alexis de Tocqueville (1805–1859) and Émile Durkheim (1858–1917), analyzed how people failed to adapt to changes in social conditions, such as greater social equality and mobility (Tocqueville) or abrupt transitions in wealth (Durkheim). In the 1930s, Robert Merton (1910–2003) was one of first to systematically differentiate between the various human responses to mismatches between what he called *“*cultural aspirations*”* (Merton 1938: 672) and the *“*differential access to opportunities among those variously located in the social structure*”* (Merton 1995, p 6).

This brief genealogy highlights that sociologists relied on two terms to explain mismatches: opportunities and aspirations. A more contemporary sociologist, Jon Elster, builds on these ideas^1^ to outline a basic framework for analyzing social behaviour in which mismatches can be described and analyzed using three basic concepts: desires, abilities, and opportunities. *“*Desires define what, for the agent, counts as best. Opportunities are the options or means that the agent ‘can’ choose from*”* (Elster 2007, p 165). Abilities refer to the capacities that people have to seize opportunities. For example, Elster highlights that the capacity for rational thinking and action is an important human ability. All the three items together should be considered necessary elements of human responses, because according to Elster opportunities only lead to a response if the actor has both the desire and ability to act (Elster 2009, p 79).

**Fig. 1:**
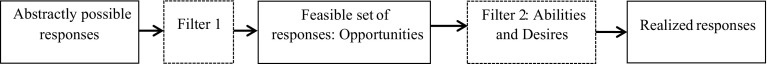
Jon Elster’s two filter model (adapted from Hedström and Udehn 2009: 34)

The way in which these three attributes work together to produce human response occurs through two successive *“*filtering operations*”*. The first filters responses that are possible in the abstract to responses that are feasible considering available opportunities, i.e., *“*all the constraints—physical, economic, legal, and others—that the agent faces*”* (Elster 2007, p 166). The other two concepts—desires and abilities—are instrumental in the second filtering operation when from this set of opportunities responses get selected that are subsequently realized by the actor (see Fig. 1).

We believe that Elster’s theory and the sociological tradition it builds on can make a useful contribution to our contemporary understanding of SE traps. The model expands the causal explanation for the occurrence of SE traps. Traps now not only originate from lack of abilities (such as adaptive capacity), they can also be produced from lack of desire, and lack of opportunities, or (more likely) a combination of all three. Yet, Elster's model also requires some additions and clarifications.

To make the model useful for the analysis of SE traps, it needs to account for the self-reinforcing effect of human response on desires, abilities, and opportunities. As explained previously, this self-reinforcing mechanism is a constitutive feature of SE traps, because it explains persistence. Elster’s analytical separation between antecedents of action (opportunities, abilities, and desires) and action itself runs the risk of missing these self-reinforcing mechanisms, which would make the model less suitable for the analysis of traps.

**Fig. 2:**
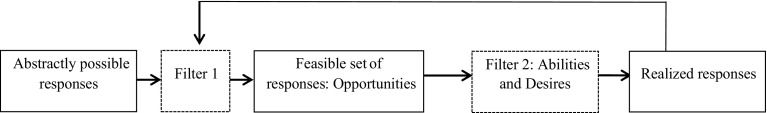
Jon Elster’s two filter model (adapted from Hedström and Udehn 2009: 34) extended with the idea of structuration (Giddens 1979; 1984), i.e., the effect of responses on opportunities

To account for self-reinforcing effects, we build on Giddens’ ideas about structuration (Giddens 1984). Structuration refers to the ongoing interaction and influence between human action and the conditions that (re)produce action. Obviously, Elster is aware of structuration effects, since he elaborates in detail the interaction between desires and opportunities (Elster 2009, pp 79–93). What is less clear in Elster’s model though is that these interactions are mediated through human responses. In some cases, Elster seems to deny this, for example, when he maintains that desires and opportunities directly influence each other. In contrast, we argue that humans ordinarily know what they (can and want to) do from practical engagement in the world (Dewey 1922; Ingold 2011). Human desire, thus does not spring from pre-given, Kantian categories of understanding but from action and response through which people are immersed in a social and ecological world (Gross 2009). Just as with desires, people’s abilities originate and develop from practical involvement in the world they inhabit. Through involvement, people acquire an embodied repertoire of habitual thought and action through which they (can) mobilize social, cultural, economic, and symbolic resources and respond to changes (Bourdieu 1986; Dewey 1922). This repertoire consists at one and the same time of deeply internalised, practical ways of acting as well as discursive, and deliberative modes of action (Giddens 1984; Haidt 2001; 2012; Vaisey 2009). Integrating structuration with Elster’s model thus means that opportunities, but also desires and abilities are both “medium and outcome of the reproduction of [human response]” (Giddens 1979, p 5). With this addition, we have established a feedback relation between (a) human response and (b) opportunities, abilities, and desires (see Fig. 2). In line with our definition, this feedback needs to be self-reinforcing for SE traps. Furthermore, to make Elster’s model suitable for the analysis of SE traps, it also needs to incorporate more explicitly how ecological processes constrain and enable human response, and how human response in turn influences ecological conditions (Fraser et al. 2003).

## A typology of traps and human responses

The simple model outlined here allows finer distinctions between the production and the reproduction of SE traps. We highlight some possible distinctions using a typology that is based on the model, and illustrated with type descriptions from earlier social science studies. This typology distinguishes between responses that can potentially contribute to the maintenance or resolution of SE traps.

In general, social science scholars have described types of responses that lie on a continuum between conformation and resistance to existing opportunities. In the first type of response, people match their desires to their opportunities. This type of response has been described as “resignation” by Elster (2009) and as “conformity” and “ritualism” by Merton (1938). In the second type, people try to change the opportunities to match their desires. Both Elster (idem) and Merton (idem) speak here of “rebellion”. Different from Elster, Merton also identifies a third type of response, which he calls “retreatism”. This type of response includes a wide range of behaviour, including acquiescence, dissimulation, foot dragging, evasion, feigned ignorance, inaction, withdrawal, and resignation (see Hirschman 1986; Scott 1985, 1990).

Based on Elster’s model and the above-cited literature, our typology includes the following five types of response: thick conformity, thin conformity, resignation, innovation, and rebellion. Table 1 gives a more elaborate description of these types. The typology primarily describes individual human response. This is due to the epistemology of methodological individualism that underpins Elster’s model (Elster 1982; Udehn 2002), but also an effect of the poor theorization of collective responses to traps, such as social movements (for an exception, see Enqvist et al. 2016, this issue). The typology indicates that response diversity to traps is influenced most by people’s abilities and desires, simply because in trap situations, the set of feasible actions are limited to the extent that only one single response option remains open. In these cases, the constraints of the first filter “are so strong that there is nothing for the second filter to work on” (Elster 2007, p 166).

**Table 1:** Typology of responses from (mis)matches between desires, abilities, and opportunities related to their effect on traps

Potential effects on SE traps	Response type	Description
Maintenance	Thick conformity	Actors have neither the ability nor the desire to change trap situations. This type of response is based on a deep cognitive acceptance of both the opportunities that are available and the actor’s abilities. Moreover, the actors’ desires also match abilities and opportunities. According to Merton (1938: 677) this type of response is *“rule rather than exception”* because without it societies and communities would suffer from inherent instability and discontinuity. It is called ‘thick conformity’ because actors do not need to deliberatively restrain their abilities. Moreover, thick conformity is characterised by both *“inwardly and outwardly”* conforming. Not only people’s actions but also their desires and abilities conform to opportunity contexts (Elster 2007: 372). The adaptation of desires and abilities to certain opportunity structures occurs habitually. Elster (2007: 175) has explained conformity as a form of *“dissonance reduction”*. Actors habitually adapt their desires to social and ecological constraints to reduce feelings of alienation and social misplacement. Norbert Elias refers in his “The Civilizing Process” (2000 [1939]) to the thickening of conformism, i.e. the social constraint towards self-restraints: *“The social standard to which the individual was first made to conform by external constraint is finally reproduced more or less smoothly within him, a self*-*restraint which may operate even against his conscious wishes”* (2000 [1939]: 109)
	Thin conformity	Actors have the ability to change SE traps, but lack the desire to do so. In this type of response the exercise of abilities is restrained, because people are in principle able to change a trap situation. However, they do not exercise this ability because they maintain desires that reinforce a trap situation. Tocqueville describes this type of response when he explains how the pressure to conform (re)produces situations, which are irrational or not beneficial for the individuals concerned. It refers to a process in which unhelpful desires become self-sustaining because nobody attacks it due to the pressure to conform. Or, in Tocqueville’s words: “[A] *powerful pressure that the mind of all exerts on the intelligence of each”* (Tocqueville 2004 [1835/1840]: 491); [this] *hollow ghost of public opinion is enough to chill the blood of would*-*be innovators and reduce them to respectful silence”* (Tocqueville 2004 [1835/1840]: 758). Elster has labelled this process as ‘pluralistic ignorance’ (Elster 2009: 40)
	Resignation	Actors have a desire to change SE traps but lack the ability to do so, and accept that this is so. This type is characterised by a mismatch between desires and abilities to change opportunities. Most scholars describe this response in very negative terms: *“defeatism, quietism and frustration”* (Merton 1938: 678); *“confusion and misery”* (Tocqueville 2004 [1835/1840]: 270); *“a source of torment to itself”* (Durkheim 1951 [1897]: 247); *“continuous individual anxiety and restlessness”* (Elster 2009: 127). Although this response type is generally perceived as negative, several scholars have also pointed to its potentialities. Scott (1992) introduced the concept of “hidden transcripts” to explore responses of peasants that hold the middle between open revolt and conformity. Likewise, Hirschman highlights the importance of passivity and acquiescence for economic development; under the right circumstances it can activate the human responses of *“exit”* or *“voice”* which in turn can trigger recovery and innovation (Hirschman 1970; 1986). When this happens resignation transforms into the type of response that is described below as innovation and rebellion. Even Tocqueville, although generally preferred desires, abilities and opportunities to be matched, was also conscious of the innovative and creative potential coming from mismatches (Tocqueville 2004 [1835/1840]: 524; 540-543). But, as Marx pointed out, potentialities are often not used due to *“the dull compulsion of economic relations”* (Scott 1985: 246), or, in other words, ‘making ends meet’. As several studies show, rebellion is typically considered as means of a last resort when people’s basic livelihood is under threat (Scott 1987; Menzies 2000)
Dissolution	Innovation	Actors have a desire to change SE traps and have the ability to do so. Innovation is the type of action that is often described in relation to the dissolution of SE traps. It refers to ways of thinking and doing as performed by so-called ‘change agents’ (Westley et al. 2013); ‘institutional entrepreneurs’ (Rosen 2014); ‘experimenters’ (Folke et al. 2003: 364); and ‘leaders’ (Olsson et al. 2004). These people have the ability—or ‘agency’ (Westley et al. 2013— to change their opportunities, and also have the desire to do so. Here it is also possible to highlight subtypes and to make a distinction between innovation that succeeds in changing trap situations, and innovation that fails to do so. The latter subtype is described by Clifford Geertz (1963) as *“agricultural involution”* (see also Authors 2014) and can readily transform into ‘resignation’
	Rebellion	Actors have a desire to change SE traps but lack the ability to do so, and do not accept that this is so. Rebellion is also a relatively well-known type of response (see e.g., Wolf 1969). Essential for rebellion is the motivation to change SE traps and the non-acceptance of the inability to do so. It is for this reason the exact mirror type of (thick and thin) conformity. As explained previously rebellion can spring from resignation and thin conformity. In contrast to conformity rebellion does not occur very frequently

The following section presents three cases of how primary resource users respond to SE traps to illustrate the empirical relevance of the theoretical model and typology presented here. Each case starts with a description of the SE trap, after which it outlines how people respond to the trap.

## Swedish archipelago and coastal fishers in the Baltic Sea

It can be argued that Swedish Baltic Sea fishers are trapped (Hammer et al. 1993; Selling and Holmer 2007; see also Kittinger et al. 2013). The economic profitability of the fishery is low (Waldo et al. 2013; Eggert and Tveteras 2007); the resilience of Baltic fish stocks is impaired due to overfishing, climate change, and eutrophication (Österblom et al. 2007; Blenckner et al. 2015); fisheries regulation has increased substantially (Hentati-Sundberg and Hjelm 2014) just as the competition from seal and cormorant populations (Königson 2011). It is, therefore, no surprise that between 1914 and 2012 the total number of professional fishers in Sweden diminished by a dramatic 93 %. This case highlights how “archipelago fishers” (Boonstra and Hentati-Sundberg 2016), who mostly work with 6–12 m boats and gillnets, meet these challenging opportunities differently in relation to the so-called ‘cod collapse’. During the 1980s, both herring and cod stocks waxed and waned tremendously (Lade et al. 2015), which led to the disappearance of local trading, processing, and retailing facilities, and at the same time, large-scale fishing operations (predominantly from the Swedish West coast) started to expand and intensify their Baltic fishery.

What follows are pen portraits of two fishers—Rune and Kenneth^2^—that were constructed from qualitative interview studies on fishing styles in the Swedish Baltic fishery performed between 2010 and 2015 (see Boonstra and Hentati-Sundberg 2016 for more information). These portraits illustrate how two archipelago fishers responded differently to a trap situation based on differences in their desires, abilities, and opportunities. Kenneth’s response resembles ‘resignation’. He had the desire to change situations for Swedish archipelago fishers and also worked to transform the opportunities offered, but in the end lacked the ability to enforce any real change. Rune’s response resembles ‘innovation’, because he also had the desire to change his situation, but in contrast to Kenneth had developed abilities that allowed him to act on opportunities.

**Fig. 3:**
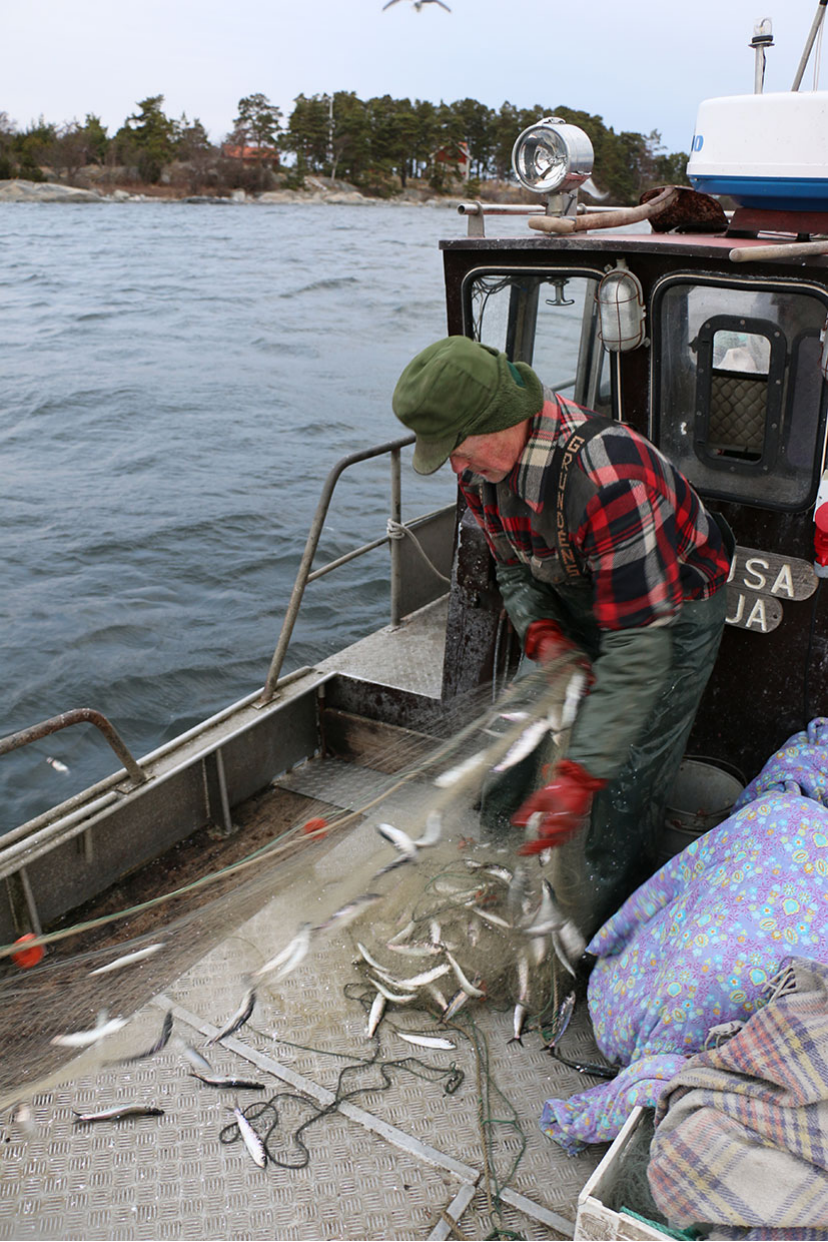
Rune at work at the Baltic Sea (Photo: Viveca Mellegård)

Rune started to fish in Stockholm’s archipelago when he was five years. His first fishing was ‘fjällfiske’—the term he uses for fishing a mixture of fresh and salt water fish, such as pike, perch, flounder, and whitefish. When he finished school at the age of 16, he began to fish herring with gillnets, but always kept doing ‘fjällfiske’ on the side, especially during summertime. The herring was sold to traders; the other species to tourists. When cod stocks began to grow at the start of the 1980s, Rune switched, such as many others, from targeting herring to targeting cod. However, unlike the other fishers, he always continued selling some of his catch directly to tourists. Rune anticipated the coming collapse and decided, together with his family, to switch strategies. Instead of specializing in cod and selling the fish to outside buyers, they started to sell all their fish to local customers. Rune developed a fishing style that targeted a broad mixture of fish (See Fig. 3). Everything was sold in their own restaurant which they opened in 1990. Rune discusses the switch:

*“*[…] *We had a very big increase in herring, which then declined and disappeared, and then there was a whole lot of cod which then flattened out and declined* […]. *Ingalill [Rune's wife] and me were a little ahead of the others because we anticipated this drop. When one fish species is on top, nothing is as sure as it will go down. Nothing helps against it. And when it is down and splashing it usually goes up again. So that was why we started with this business. It was the 1990s. And it was when it started to drop we said “this is not possible we need to find another direction” and so we started with that. We had a small farm shop during a couple of years so it was only for us to start expanding this farm shop and start placing some tables, and then there was beer and some potatoes. In this way we expanded slowly but surely. So when the others struggled with herring and cod we were already into the next fishing that we based our business on. And that’s what gave us this business.”*

Kenneth too has been an archipelagic fisher all his life, and just as Rune, he targeted a mixture of herring, cod, eel, salmon, and freshwater species, such as pike and perch. Nevertheless, Kenneth relied mostly on herring and cod for his income. After a long career as a fisher and representative of the local fishers’ union, Kenneth is now semi-retired and fishes only occasionally. He is pessimistic about the future of archipelago and coastal fishery. This pessimism grew from his daily experience with strict and complex fishing regulations, seals eating his catch, the closure of local fish-processing facilities, and the competition with offshore trawlers over herring and cod. As a representative of the local fishers’ union, he found that politicians, scientists, and the general public ignored the voice of Baltic archipelagic fishers. For example, Kenneth and his colleagues argued for restricted trawling during the cod boom but no one listened:

*“*[…] *We had incredible fishing during some years. Something that you in your wildest dreams never could think of. But what also happened then was that the whole Danish North Sea fleet came* [to the Baltic], [which is why] *we argued that there has to be a restriction because this is an inland sea. But we were only a voice in the wilderness and not heard in practice. They just said “well you don’t have to scream for so long because when all the cod is caught we* [Danish and Swedish offshore trawlers] *will not be here* [any longer]*, but that was not* [the answer that] *we wanted. But when it comes to governments we have had incredible difficulties with getting our voice heard”.*

His efforts to improve the opportunities of archipelago fishers by influencing fisheries regulation remained fruitless, which made him bitter and frustrated: “[…] *So we do not know how long we should keep going, it feels pointless. You think that you would be able to sail in headwind, but that is not really possible*.” As a consequence of these experiences, Kenneth discouraged his son from becoming a fisher.

The fact that these two fishers continue to practice that their trade is testimony of their abilities and desire. According to Rune, it is *“difficult almost impossible”* to describe a skilled fisher:*”You have to* […] *manage both one thing and another within fisheries. Everything from currents, wind and water temperature, yes everything like that matters, where to fish, how to fix gear and place new gears and… So it is something you can’t learn over one night’’.* Kenneth highlights similar abilities, when he talks about the expertise and skill involved in handling “*motors* [and] *technologies*”, but also having embodied skills:“[so it’s] *innate, when you interpret weather and wind and so on.*” Nevertheless, their ability also differs. Over the years, Rune and his family developed the ability to clean, cook, and sell fish to customers on markets, directly at their house, or in Stockholm. The skills and knowledge learned through these experiences allowed him to become less dependent on the local seafood industry and trade.

Understanding desires that lie behind their life-long archipelago fishing is not so straightforward. Kenneth compares his fishing with “*narcotics*”—and also highlights that there is the aspiration of wanting to “*succeed*” in the competition with others—“*To find the best places and be the worst* […]. *You don’t want to be the crappiest when you are out, you want to be the best*”. Rune highlighted in this respect that their family was one of the last at their island who desired to maintain a more or less self-sufficient livelihood based on fishing and small-scale farming. He commented that many of his colleagues quit farming and began working as carpenters and builders. “*It was like the others didn’t have the ability any longer. And many of my old colleagues started with other work, so it was a little too complicated to work with animals as well*”.

## AmaXhosa rural dwellers in the Eastern Cape, South Africa^3^

In the former Transkei homeland, in the Eastern Cape province of South Africa, many households are poor and there is a high rural population density. In the Mqnuma Local Municipality, where this case study was conducted, population density reaches 77.18 people/km^2^ (calculated from Census 2011 Community Profile Database). Many inhabitants engage in some form of cultivation but produce is supplementary to other livelihood strategies, and there is a heavy reliance on welfare grants (Hebinck and Lent 2007). Reliance on agriculture for livelihood security is surprisingly low. Only 6 % of households with access to land raise income from it, which means that most households rely on store-bought maize meal throughout the year (du Toit 2005). During the Apartheid era, people of this region used to practice small-scale cultivation and livestock farming. These rural livelihoods were often complemented with financial inputs from migrant family members working in the mines or cities outside the rural homelands (McAllister 2001).

However, the region has seen the long-term decline of cultivation and livestock husbandry (Beinart 1992; Ainslie 2005; Andrew and Fox 2004; De Klerk 2007), resulting in widespread abandonment of arable fields and gardens, particularly during the transition to democracy (Andrew and Fox 2004; De Klerk 2007; Shackleton et al. 2013). The decline is attributed to insufficient inputs. According to Shackleton et al. (2013), the poorest people in the Transkei cannot afford to cultivate fields, because they do not have access to capital, equipment, or farming inputs. Inhabitants point this out too when they use the Xhosa phrase “*kunzima ukulima, kuyasokolo*” (it is difficult to cultivate, because ‘we are suffering’ financially). After Apartheid ended, the formal migrant labour system collapsed forcing migrants from the Eastern Cape to take up poorly paid short-term work (Bank and Minkley 2005; Cox et al. 2004; Todes et al. 2010). The drop in remittances sent home (Hebinck and Lent 2007) meant that peasants were unable to purchase agricultural supplies and livestock and to invest in homestead infrastructure (McAllister 2001; Masterson et al. 2014). Moreover, the new democratic government also decreased the support for livestock farming (Everatt and Zulu 2001) which resulted in reduced animal draught power in this region (Shackleton et al. 2013).

**Fig. 4:**
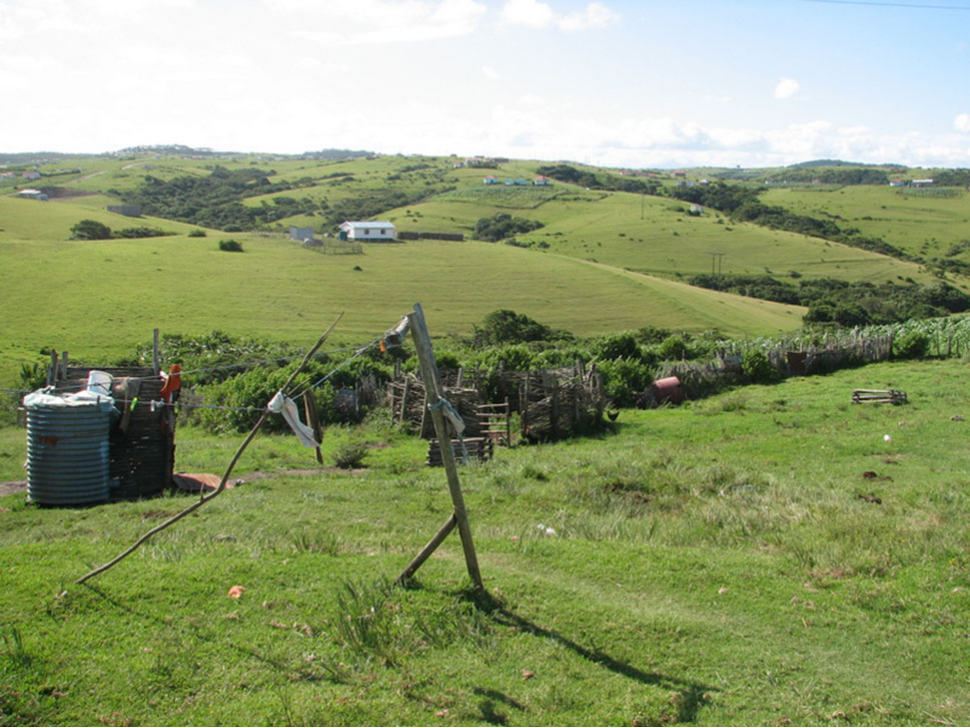
Small home-gardens at homesteads built within larger abandoned fields. In the foreground a cattle-byre is empty

Abandonment of fields has over the years resulted in land cover changes with encroachment and substantial forest revegetation (Shackleton et al. 2013; Chalmers and Fabricius 2007; De Klerk 2007). Abandoned fields are colonized by *Acacia* species, which over 40–50 years gradually give way to forests (Shackleton et al. 2013). Dense stands of thorny *Acacia* sp. make grazing difficult, and require labour-intensive clearing if the fields are to be ploughed again. Inhabitants point out that once these woody species get too large and dense the cost of removal becomes too high, precluding a return to cultivation which further marginalizes livelihood security and keeps rural dwellers trapped in poverty (See Fig. 4).

Using the concepts introduced earlier, we can identify several responses to this SE trap. First, inhabitants have a desire to cultivate land and keep animals, because ploughing and owning cattle affords status in the community. Or as one respondent phrased it: “*You lose your dignity if you don’t plough. Amongst other men your ideas won’t be taken seriously, because they take you as somebody who is irresponsible; even your ideas are not important.*” (Thembalani^4^ 63 years). People receive respect and gain independence through building a homestead, cultivation, and livestock ownership. With their farm labour, they become a “responsible Xhosa”; a person who provides food for one’s family and pays homage to one’s ancestors through rituals (which require agricultural products, such as maize for traditional beer, and may include sacrificing livestock).

It has become harder for people to meet these desires, because it requires that people own land and can invest money to provide inputs for the land, such as draught power and fertilizer. Often people are not able to do so. To own land, young men first need to marry. In addition, for a marriage, they need a bride price, or “*lobola*”, in the form of cattle or a lump sum. To obtain *lobola*, these men work in the cities or mines. As explained previously, job opportunities in the cities or mines for migrant workers have significantly decreased since the end of Apartheid. In other words, these men have the desire to cultivate and keep cattle, but lack the ability to do so. In what follows, we present the response of two young men, Siyabulela (32 years) and Thozamile (20 years), which exemplify the responses of many more young men in the Eastern Cape. Using the typology, their response comes closest to ‘resignation’.

These men have been through the “*Ulwaluko*” rite of passage and have been initiated into manhood. However, to fully be considered men, they must have their own homestead and marry for which they need a bride price. An income would enable them to acquire these things. As Siyabulela explains:

*“*[My friend] *is stressed because he has to do everything for himself because he has no*-*one to assist him.* […] *He’s not married because he is not working. To have a wife, the first thing you have to do is to work and have some money, and some cattle to pay a bride*-*price for that.* [My friend] *has long been striving to get some work in the city, but with no luck. He decided to come back* [to the Transkei] *and stay at home. Because there in the city, no*-*one will be able to help him. His mother is still alive and earns a pension grant. He doesn’t feel good to depend on his mother’s pension grant because he is the son, who is supposed to look after his mother, not* vice versa*, but circumstances do not allow him to”*.

When these men return to the rural villages without employment or income, they fail to meet expectations and are not able to attain the independence and respect that comes with employment, a homestead, livestock, and marriage. Often this outcome is attributed to the will of the ancestors: for example, a particular ancestor requires that a ritual be performed. However, a traditional ritual that would expedite favourable outcomes from the ancestors requires significant financial resources, including the slaughter of an ox (which may need to be purchased), food and beverages for all the clan members, and transport costs. As Thozamile explains, this is expensive:

*“There is a home*-*garden at my home, it is planted. I am the one who has done that. There are goats at home, and I also look after them. It’s not what I want to do, myself. I want to have some work, and provide for the family: to be able to have livestock and be able to plough. But because I’m amongst the homesteads and not at work, I am responsible for that* [the garden and goats]*. The problem is I am still depending on someone. I want to be independent. To be independent I must have a job, get some money, do whatever I can at my home: build structures, and also have livestock to perform the rituals. Because even now, I think that it might be the problem with me* [my struggle to find a job] *because I haven’t performed the* [correct] *ritual. I cannot afford the ritual”*.

The short stories of Siyabulela and Thozamile (and their peers) can be contrasted with Sandile’s history. During the 1990s, he had short spells of employment in construction companies, and as an electrician, in Cape Town, but he returned to his village several times due to unemployment. In 2003, he moved back to the village in the Transkei for good to live with his wife and children:

*“That was the period that I made a decision that I am interested to stay and make a living here* [at home]*. Without enough skills, work is scarce for you. But for me there were limited chances to get a job. I have skills in various things, but the problem is* […] *I do not have the qualifications.* […] *I went to the city, to work with the objective of getting some capital quickly, so that I could come back home and start earning here. Because I am someone who doesn’t like city life. I enjoy being at home, and farming. The climate in the city is not good for me. Here in the country I like the air.’*

In Sandile’s case, he had both the desire and the abilities, due to the money earned in Cape Town and his skills as an electrician and builder, to take up farming at the family homestead, ploughing the family fields, and rearing relatively large herds of goats, sheep, and cattle. He also experiments with intercropping and adapting planting seasons for cabbage, maize, and potatoes. Additionally, he has also started to keep bees. Through observing and recording the flowering times of different trees, he places his hives in such a way that the bees can reach both winter and summer flowering trees. It ensures him of honey harvests all year round. Moreover, with the skills learned as electrician and builder, he also earns some extra income serving rural families. Being able to do so, makes his response qualitatively different from the resignation in Thozamile’s and Siyabulela’s stories. Sandile’s response, especially how he develops his homestead, resembles much more the response that we described in our typology as ‘innovation’.

## Smallholders in the Pamir Mountains

The Pamir Mountains of Eastern Tajikistan, bordering Afghanistan to the South, China to the East, and Kyrgyzstan to the North, are home to an isolated population who have subsisted for millennia on ‘combined mountain agriculture’, a combination of transhumance and high-altitude agriculture (Kreutzmann et al. 2010). Despite this isolation, the region has been subject to various external interventions due to its geopolitical significance as a border region (Hopkirk 1994).

During the time that the Tajik Pamirs were part of the USSR, the remote and independent society was transformed through Soviet agricultural reforms. Ancestral communal land was consolidated into collective and then state farms, and Pamiri people were appointed jobs as farm labourers, industry workers, schoolteachers, or doctors (Bliss 2006). Fodder was flown in by helicopter to high pastures to ensure that the growing number of livestock could be fed.

Over time, these agricultural reforms completely failed as the pastures became quickly denuded (Bliss 2006), biodiversity declined (Giuliani et al. 2011), and deforestation caused soil erosion and desertification (Herbers 2001; German Technical Cooperation 2012). Deforestation and soil erosion in turn increase the impact of natural disasters, such as avalanches and landslides, which also occur more frequently as a result of rapidly melting glaciers in the Pamir Mountain range (Armstrong 2010). The situation worsened when Tajikistan fell into civil war following the collapse of the Soviet Union. The state farms dissolved, many livestock died, and the factories abruptly stopped operating.

During much of the 1990s, the Pamiri were isolated again, but this time unable to self-provide due to the population growth and lack of ability to farm the land. For their basic needs, people relied almost solely on external humanitarian assistance. Since then there has been a constant and increasing flow of goods and projects into the Pamirs. The World Bank, for example, has started a Cadaster programme (World Bank 2015) to register the land rights of Pamiri people, agricultural extension services have been set up, and every year, new seeds and fertilizers are introduced (Haider and Van Oudenhoven 2015). Despite this history of interventions, well-being in the Pamirs remains relatively low; it is considered as the poorest area of Central Asia (Food and Agriculture Organization 2015). The SE trap in the Pamirs consists of poverty that has become persistent through self-reinforcing feedbacks between structural and long-term dependence on external interventions, degradation of natural environments, and a history of emigration. To uncover strategies to dissolve the trap, a deeper look at human responses is helpful.

Using the concepts introduced earlier, it is possible to say that Pamiri desire to stay on the land, both as a result of an affinity to the landscape, but also because of a spiritual and religious feeling of loyalty to the Pamirs on account of the Aga Khan, the spiritual leader of the Ismaili Muslims (nearly 99 percent of people in the Pamirs are Ismaili). A young schoolteacher in Bartang Valley who spent three years working in a street kiosk in Moscow described her desire to stay in the Pamirs when she returned:

*“If there would be a choice, I would never leave… I would live here. It’s the best place to live. Whenever I dreamed, I was never in Moscow. My dreams were here [in the Pamirs], where I’m climbing the mountains, or I’m crossing the bridge to the next village. I’m always here, always here, nowhere else.”*

Although there still is a strong desire to stay in the mountains, for spiritual and cultural reasons, many people lack the abilities to do so. A whole generation of farmers has been lost as people were forcefully taken off the land during the Soviet era, and trained to be teachers, doctors, or factory workers. Over the years, poverty reduction efforts focused primarily on mechanization and externalization of farm labour. The most frequent response for people from this generation and later ones is to emigrate in search for work.

As a consequence of Soviet rule and two decades of intensive humanitarian assistance, Pamiris have come to depend on external inputs through external assistance or remittances. The majority of young men, and many young women, work in Kazakhstan and Russia sending money home. Tajikistan is the most remittance-dependent country in the world, with more than half of its GDP coming from remittances (United Nations Development Programme 2014). This type of response can be labelled as resignation: a desire to change the SE trap but lacking the ability to farm and, therefore, leaving. A farmer from Chidz, a village in the valley of Rushan, describes this situation as follows:

*“We became lazy because we received everything* […]. *We became dependent on Soviet fuel and we are still dependent today. When we have a problem, we go and look for a development agency and ask for help. We feel powerless, because we have become linked to a global system which is entirely out of our control. If oil prices go up, we suffer. If Moscow hits a recession, we feel it here.”*

**Fig. 5:**
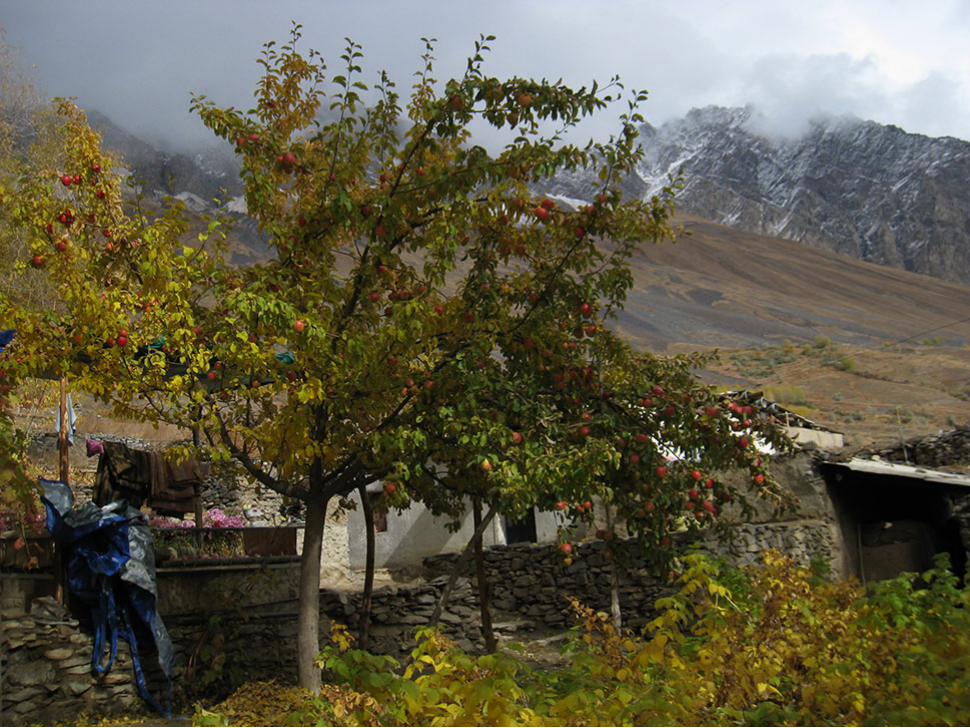
Apple trees in Dowlatman Mirasanov’s garden in the Pamirs (Photo: Frederik van Oudenhoven)

Just as we have seen in the previous cases there are also individuals in the Pamirs who for some reasons manage to stay in the mountains to successfully farm. They have a desire to stay and farm, and also maintained the ability to do so. In the typology outlined previously, this type of response is labelled as ‘innovation’. Dowlatman Mirasanov lives in the valley of Rushan. High above the village, he grows a variety of fruit trees on vertiginous slopes, where one would assume nothing at all could grow (See Fig. 5). Conventionally, fruit growers take cuttings from a parent tree with desirable qualities and graft them onto an established rootstock that is well-adapted to local soil conditions. This is done to ensure that new trees grow and that fruits keep these desired qualities. However, Dowlatman prefers to grow fruit from seed directly, so that with every seed that sprouts, a truly new tree will develop, and one combines the characteristics of its parents to yield something unexpected. Sometimes, the unexpected is disappointing, but it can also mean the birth of a new fruit variety, or a tree which adapts particularly well to the challenging conditions in the mountains. Living proof of the potential of this very ancient method of plant breeding is the apple variety to which he bestowed his own name: the Dowlatmani. It is the size of a grapefruit, crunchy, and with a spectacular sweetness and intensity of taste. Most farmers in Rushan valley, Dowlatman claims, grow at least one or two varieties originating from his garden (see Haider and Van Oudenhoven 2015, p 264).

## Discussion

Conventionally, the occurrence of SE traps is mostly attributed to a single factor: lack of adaptive capacity (Carpenter and Brock 2008; Scheffer and Westley 2007). The exclusive attention to adaptability results in an ‘all or nothing’- categorization (see Goertz 2006: 29) of responses. People having adaptability are able to avoid or dissolve SE traps, and those that lack it maintain them.

With the typology introduced in this paper and illustrated empirically, we highlight that more factors can and should be used to understand the diversity in behavioural response to SE traps. By adding desires and opportunities to the explanatory mix, it becomes possible to differentiate human responses to traps in more detail. This is important because, as we demonstrated here, under some conditions, lack of adaptability is not a causal factor producing SE traps. In these cases, one has to search for other factors of importance, or interactions between various factors, including ability, desires, and opportunities.

This framework also opens up for research questions that focus on understanding the feedback between response and opportunities, feedbacks hold the key for avoiding or dissolving SE traps. Using Giddens’ structuration theory, we conceptualised how different response types can potentially expand, maintain, or reduce the opportunity set, i.e., the set of responses feasible considering all social and ecological constraints (see Fig. 2). It is important to add a disclaimer here. As we have written throughout the article, individual responses have a potential to resolve traps. Whether or not this potential is realized will depend on how individual responses interact with other responses, and together influence social and ecological conditions that determine opportunities. This interaction is causally complex and typically emergent, i.e., patterns of social-ecological behaviour, which are collaboratively created from individual responses, but which are not reducible to those responses (Sawyer 2005). We are fully aware that this article has only begun to bring this knowledge in dialogue with the theorization of SE traps. Explanation and conceptualization of the feedback process between response and opportunities (or between ‘agency’ and ‘structure’) are one of the major themes in the social sciences (for an overview, see Giddens 1984; Sawyer 2005; Elder-Vass 2010). There, thus, exists a wealth of knowledge that can be used to further explore this feedback relation and to better understand why and how SE traps are transformed or reproduced. One important direction for future research where this knowledge can be instrumental is the development of different intervention strategies for avoiding or resolving SE traps. Based on the argument presented in our article, we hypothesise that the response types which maintain traps would need to be targeted differently in policy and planning. A good start would be to consider in more detail how desires, ability, and opportunities have been theorised so far and how scientists attribute causal force to these aspects. Without having any pretension to be inclusive, we would like to point to some ways in which social scientists conceive of the three concepts that we used in this article: desires, abilities, and opportunities.

It is often assumed that opportunities are the factors that have the greatest explanatory force, such as in the proverb ‘opportunity makes the thief’. Indeed, in Economics, it is assumed that all people have essentially the same desires (e.g., maximise pleasure and avoid pain) and that only opportunities differ (Stigler and Becker 1977). This view also holds sway in Political Science and Administrative Science, e.g., in the idea of ‘nudging’ (Thaler and Sunstein 2008), whereby social and individual changes are accomplished by intervening in people’s opportunity set.

Ability as an explanatory factor also has a rich theoretical tradition. At first, it seems to readily equate to adaptability and to operationalise as control over natural resources, technology, money, or knowledge (Carter and Barrett 2006). However, lack of adaptability includes more than a lack of material resources. They also exist due to lack of bridging and bonding social relations (Woolcock 1998), lack of self-control and mindless choosing (Thaler and Sunstein 2008), lack of ideas (Haider and van Oudenhoven 2015), or from the belief that one cannot determine his or her own fate (Lefcourt 1982). Here, we suggest operationalizing ability using the social science literature on agency and power (for overviews on these concepts, see Emirbayer and Mische 1998 and Boonstra 2016 respectively).

The final explanatory factor for traps—desires—is perhaps the dark horse of the three. Desires are clearly less well described in the literature, probably since they are difficult to observe, and because desires were for a long time narrowed to ‘interest’ as the only motivation driving human choice and action (Hirschman 1977; Force 2003).

## Conclusion

As this special issue showcases, social scientists are coming to grips with the concept of social-ecological traps. The objective of this paper, to explore a finer distinction of human responses to SE trap situations, aligns with these efforts. The paper first introduced a conceptual model to outline the interrelations between desires, abilities, and opportunities. It then used the model together with social science literature to construct a typology that distinguished between five types of responses to SE trap situations: (1) Thick conformity; (2) Thin conformity; (3) Resignation; (4) Innovation; and (5) Rebellion.

The empirical relevance of this model has been illustrated with three cases: Swedish archipelago fishers in the Baltic Sea; amaXhosa rural dwellers in the Eastern Cape, South Africa; and smallholders in the Pamir mountains of Tajikistan. Our article demonstrates how differences in abilities and desires translate into different ways of responding to trap situations. It also highlights how these types of response influence social and ecological conditions, and can change opportunities for the people involved. These results emphasize that it is possible and important to pay attention to the diversity of responses in relation to the understanding and possible resolution of social-ecological traps.

## Footnotes

1 Elster demonstrates how this basic model is indebted to Tocqueville (Elster 2007, 2009). For Elster’s assessment of Merton see Elster (1990).

2 The name of this fisher has been anonymized.

3 This case is part of a larger study on the perceptions of agriculture and place in three villages in the *Mnquma* District, former *Transkei* homeland. The study was performed using interviews, focus groups and participatory photography (Masterson et al. 2014).

4 Names have been anonymized.
